# Plasmapheresis without apparatus in complex care of victims with crush syndrome during the first hours after extrication in a field hospital of EMERCOM of Russia in emergency areas

**DOI:** 10.1186/cc10981

**Published:** 2012-03-20

**Authors:** A Popov, I Yakirevich, A Skorobulatov, V Shabanov

**Affiliations:** 1 EMERCOM of Russia, Zhukovsky, Moscow Region, Russia; 2 All-Russian Centre of Disaster Medicine, Moscow, Russia

## Introduction

This is the generalization of the experience of membranous plasmapheresis without apparatus (MPPA) application in the complex care of victims with crush syndrome (CS) in the field hospital (FH) of EMERCOM of Russia during elimination of medical consequences of earthquakes (Pakistan, 2005; China, 2008; Haiti, 2010).

## Methods

Thirty-eight victims with CS (19 males, 19 females, age 34.5 ± 4) were in the resuscitation department of the FH. Compound fractures of tubular bones and crushed tissues necrosis were observed. Joint movement was severely restricted and artery pulsation was uncertain. Condition severity: according to the Glasgow Coma Scale 12 ± 1, according to APACHE II score 29 ± 4. The tendency to hypotension and tachycardia, increase of body temperature and dyspnea intensification were observed, diurnal diuresis decreased. Plasmapheresis treatment was carried out by the MPPA method. A total of 2 ± 1 procedures were conducted to each patient with the removal of 70 ± 10% of the plasma circulation volume per session. Removed plasma volume was calculated for each victim individually on the basis of average volume before plasma exchange. The procedure frequency was once per day. Substitution means: crystalloids, hydroxyethylized starch, proteins. The MPPA procedure time was from 60 to 120 minutes. MPPA was carried out in all victims during complex care for CS: elimination of painful impact and stressful situation; restoration of acid-alkaline conditions and water-electrolytic balance of blood, maintenance of hemodilution with 25 to 30% hematocrit; correction of the blood coagulation system; detoxication with the application of active methods of homeostasis correction; prevention and elimination of purulent and septic complications; primary surgical debridement and excision of necrotic mass areas carried out with general anesthesia, no excision conducted; and transport immobilization before evacuation.

## Results

Among all victims, hemodynamics stabilization was noted in 28 ± 6 hours, and dieresis increased up to 1,200 ± 100 ml/day in 18 ± 8 hours. Acute renal failure cases were not noted. All victims in stable condition were evacuated to specialized hospitals by helicopter. No mortality rate during medical aid rendering was noted.

## Conclusion

MPPA application allows one to reduce the rate of complications and mortality. MPPA application is the method of extracorporeal homeostasis correction option for victims with CS in a FH in emergencies.

